# Multimorbidity or polypharmacy: two sides of the same coin?

**DOI:** 10.15256/joc.2015.5.51

**Published:** 2015-04-24

**Authors:** Carol Sinnott, Colin P. Bradley

**Affiliations:** ^1^Department of General Practice, University College Cork, Cork, Ireland

**Keywords:** Comorbidity, chronic disease, patient care management, polypharmacy, primary health care, information sharing

Polypharmacy, broadly defined as the chronic co-prescription of several drugs [1], has long been recognized as problematic. The greater the number of medicines a patient takes, the greater the risk of adverse effects of any one medicine, and the greater the risk of drug–drug interactions. Thus, polypharmacy is an accepted risk for poor health outcomes, including hospitalizations and mortality [2]. The number of drugs per patient (or polypharmacy) has come to be used as a measure of potentially hazardous professional behaviour, and is sometimes used in conjunction with the term ‘inappropriate prescribing’. Nonetheless, the prevalence of polypharmacy is rising inexorably [3]. This rise is driven, principally, by the rising prevalence of multimorbidity, i.e., the co-occurrence of two or more chronic long-term diseases or conditions in one patient [4]. The issue is compounded by clinical guidelines that advocate the use of several medicines in the management of individual diseases and their associated risk factors [5]. The result is a dilemma for prescribers: on the one hand there is the need to keep the number of medicines to a minimum, while on the other ensuring that the patient receives what evidence-based guidelines advocate as being in their best interest [6].

Given the obvious overlap between the concepts of polypharmacy and multimorbidity, it is striking that much of the research on these two issues is conducted in parallel, rather than in collaboration. In view of this, a meeting on medication optimization in multimorbidity was convened at University College Cork in Ireland to bring together leading national and international researchers in the fields of medicines management and multimorbidity. Representatives from the disciplines of general practice (GP), pharmacy, clinical pharmacology, behavioural science, and health services research were present. Dr Martin Duerden, co-author of the King’s Fund report on ‘Polypharmacy and medicines optimisation’, was one of the speakers [7]. Using data from the report, Dr Duerden showed that there were three-fold increases in the numbers of patients receiving five or more medications between 1995 and 2010. The oldest patients were most affected, with 16.4% of those aged 65 years and older receiving 10 or more medications. However, he acknowledged that older patients with multiple illnesses had much to gain from what he termed ‘appropriate polypharmacy’, or the use of multiple medicines to achieve maximum benefit with the least risk of harm. To limit potential harm, Dr Duerden suggested identifying the patients at greatest risk of adverse outcomes from polypharmacy and tapering their drug regimens accordingly. He also emphasized the need to provide doctors in training with appropriate prescribing skills, which would include how to identify those at greatest risk and how to treat their multiple conditions more selectively.

Professor Stephen Byrne, School of Pharmacy, University College Cork, also focused his presentation on the use of medicines. He described the ongoing SENATOR (Software ENgine for the Assessment & optimization of drug and non-drug Therapy in Older peRsons) trial [8], in which he and colleagues from across Europe are developing and testing a decision-support engine to guide prescribers on how to minimize inappropriate prescribing and polypharmacy in a cost-effective way. Dr Barbara Clyne and colleagues also examined web-based treatment algorithms to guide medication review in the OPTI-SCRIPT trial [9], but incorporated this with academic detailing by a pharmacist as well as patient information leaflets. This multifaceted approach was effective in reducing potentially inappropriate medications in patients with polypharmacy in primary care.

Other researchers, using a multimorbidity perspective, focused on the qualitative nature of the patients’ morbidities, their experience of treatment burden, and their priorities around symptom control and active disease management. Here, medications were viewed as part of a wider range of issues facing patients and clinicians. Professor Susan Smith from the HRB Centre for Primary Care Research and the Royal College of Surgeons in Ireland, presented work from an exploratory randomized controlled trial, the Optimal study [10]. Findings from this study indicated that an occupational-therapy-led self-management programme improved the quality of life for people in the community with multimorbidity. Professor Stewart Mercer, from the University of Glasgow in Scotland, discussed the striking social gradient of multimorbidity. His research group have shown that the age of onset of multimorbidity is 10–15 years earlier in people living in the most deprived compared with the most affluent areas [11]. In the CARE PLUS trial, Professor Mercer and colleagues examined a ‘whole-person’ intervention in the management of patients with multimorbidity living in deprived areas in Scotland [12]. They found that additional consultation time, continuity with healthcare professionals, and the provision of supportive information led to positive effects on patients’ quality of life and well-being.

Nevertheless, there are still real challenges for prescribers who are faced with the rising numbers of medicines indicated for their patients with multimorbidity. Studies undertaken at University College Cork have highlighted the conflicts that can arise between evidence-based practice and patient-centred care in multimorbidity [13]. These conflicts lead to ‘satisficing’: delivering care that is deemed satisfactory and sufficient given the particular complexities of a particular patient. For many prescribers, satisficing means maintaining the status quo of long lists of medications. For others, it involves relaxing targets for disease control, negotiating compromise with patients, or using hunches on the best course of action to take. These clinical dilemmas may be mitigated by emerging data on the relationship between the number of medications prescribed and multimorbidity. Dr Rupert Payne from the University of Cambridge, England, another co-author of the aforementioned King’s Fund report, illustrated this by discussing how prevalent conditions such as ischaemic heart disease and diabetes are unavoidably associated with multiple medications [14]. He elaborated on how the resulting polypharmacy can actually reduce the risk of hospitalization if it emanates from appropriate treatment of multiple chronic diseases [15].

Evidently, optimizing medication management and delivering patient-centred care in multimorbidity are not mutually exclusive. Dr Molly Byrne, of the Health Behaviour Change Research Group at NUI Galway in Ireland, suggested that the key to combining these two activities may lie in the use of behavioural theory. Models of behaviour, such as those described by Michie et al., may help to understand the difficulties that arise for healthcare professionals trying to meet these dual challenges [16].

An example of an intervention trying to achieve both goals is the 3D study, which was presented by Dr Mei See Man from the University of Bristol, in England. The 3D study addresses three aspects of multimorbidity care: (i) dimensions of health (encompassing patient-centred care and quality of life issues), (ii) depression, and (iii) drugs [17]. The latter component utilizes a community pharmacist review to simplify drug regimes, assess patient safety, and address adherence, which is seamlessly integrated into the overall case-management plan.

This inaugural meeting has provided a useful platform for sharing understandings between the diverse disciplines most involved in optimizing medications in multimorbidity. It is clear that patients with multimorbidity stand to benefit from appropriate prescription of multiple medications, but these patients also experience combinations of diseases, interactions, and symptoms that preclude strict adherence to single-disease guidelines. The line between what is appropriate and inappropriate prescribing in multimorbidity may only be drawn at the level of the individual patient. The delivery of safe, effective, and efficient healthcare for people with multiple chronic diseases presents a global challenge for clinicians, researchers, and policy makers. It is an issue that goes beyond simple medication management alone, and will not be resolved by one discipline or a single research perspective. Multimorbidity and polypharmacy may well be two sides of the same coin: bringing the two research perspectives together will generate synergies and new understandings that will maximize the benefit for patients with multiple chronic diseases.
